# Use of Remote Monitoring to Improve Outcomes in Patients with Heart Failure: A Pilot Trial

**DOI:** 10.1155/2010/870959

**Published:** 2010-05-19

**Authors:** Ambar Kulshreshtha, Joseph C. Kvedar, Abhinav Goyal, Elkan F. Halpern, Alice J. Watson

**Affiliations:** ^1^Graduate School, Rollins School of Public Health, Emory University, 1518 Clifton Road NE, Room 467, Atlanta, GA 30322, USA; ^2^Center for Connected Health, Partners Health Care, Boston, MA, USA; ^3^Harvard Medical School, Massachusetts General Hospital, Boston, MA, USA; ^4^Emory Rollins School of Public Health and Emory School of Medicine, Atlanta, GA, USA; ^5^Department of Statistics, Institute of Technology Assessment, Massachusetts General Hospital, Boston, MA, USA

## Abstract

Remote monitoring (RM) of homebound heart failure (HF) patients has previously been shown to reduce hospital admissions. We conducted a pilot trial of ambulatory, non-homebound patients recently hospitalized for HF to determine whether RM could be successfully implemented in the ambulatory setting. Eligible patients from Massachusetts General Hospital (*n* = 150) were randomized to a control group (*n* = 68) or to a group that was offered RM (*n* = 82). The participants transmitted vital signs data to a nurse who coordinated care with the physician over the course of the 6-month study. Participants in the RM program had a lower all-cause per person readmission rate (mean = 0.64, SD ± 0.87) compared to the usual care group (mean = 0.73, SD ± 1.51; *P*-value = .75) although the difference was not statistically significant. HF-related readmission rate was similarly reduced in participants. This pilot study demonstrates that RM can be successfully implemented in non-homebound HF patients and may reduce readmission rates.

## 1. Introduction

Heart failure affects over 5 million people in the US, and its incidence and prevalence are rising rapidly, despite advances in Heart Failure (HF) therapies. Hospitalizations for HF have nearly tripled in the last three decades, and it is now the most common cause of hospitalization in the US among the elderly [1, 2]. In addition to the substantial morbidity and mortality, the costs associated with HF care (in particular HF hospitalizations) are $37.2 billion [3, 4]. Therefore, novel approaches are essential to reduce the morbidity and costs associated with HF hospitalizations.

Although two decades of research underscores the importance of evidence-based pharmacologic management of HF patients, it is now appreciated that non-pharmacologic interventions can also significantly influence HF outcomes [5]. A growing body of evidence suggests that non-pharmacological interventions implemented by multidisciplinary teams across the inpatient and outpatient continuum can reduce hospitalizations and/or deaths in HF patients [6, 7]. An innovative care delivery model that has recently shown promise is the use of remote monitoring (RM) technology. Remote monitoring programs acquire and securely transmit data on patients' HF signs and symptoms to health care teams, alert providers to the early signs of clinical deterioration, and create opportunities for timely intervention. RM also involves patients in their own care and allows them to link behaviors and their consequences (e.g., nonadherence to medications and subsequent weight gain) [8, 9].

Recent meta-analysis has concluded that RM programs for HF patients reduce hospital admissions and mortality and simultaneously improve health related quality of life [10–12]. Prior studies have demonstrated that RM of homebound HF patients significantly reduced home visits by trained nurses and reduced hospital readmissions [13, 14]. It is not known, however, whether the benefits of RM extend to ambulatory, non-homebound HF patients. The objective of this pilot study was (1) to determine whether RM could be successfully implemented in non-homebound HF patients, (2) to assess satisfaction with RM among ambulatory HF patients, and (3) to obtain preliminary estimates of the 6-month hospital readmission rate between non-homebound patients who participated in an RM program and patients who either declined (non-participants) or were not offered the opportunity to participate (control).

## 2. Methods

### 2.1. Study Design

**Figure 1:**
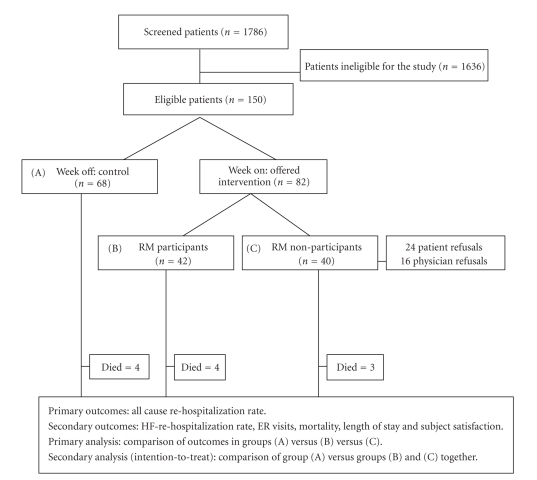
Study flow diagram. RM: Remote Monitoring; HF: Heart Failure; ER: Emergency Room visits.

A daily chart review of patients hospitalized with HF at Massachusetts General Hospital (MGH) was conducted between July 2006 and June 2007. Eligibility criteria for study participation included all of the following: current admission or recent discharge (within prior 2 weeks) from MGH with a primary diagnosis of HF, considered high risk for readmission (history of hospital readmissions for cardiac-related reasons or ejection fraction ≤20%), non-homebound, age over 18 years, not awaiting cardiac or renal transplant, English speaking, mentally competent (or willing primary caregiver), a working telephone line with a 3 prong electric outlet, and a Partners-affiliated physician or cardiologist (Partners HealthCare is an integrated health care system founded by Brigham and Women's Hospital and Massachusetts General Hospital in Boston). Eligible patients were prospectively randomized on a week-on and week-off basis either to a group that was offered RM or to a control group that was not offered RM; that is, participants were allocated to a different group each week (Figure 1). Physician agreement was also required. Patients who were offered RM but who refused RM or for whom physician approval could not be obtained were analyzed as a separate arm of the study (non-participants). RM participants received home monitoring for six months after hospital discharge and the control and non-participant group received standard care for a similar time period. All patients were followed for a period of six months from the time they were identified. The study was approved by Partners Institution Review Committee. The program was offered free of charge to eligible patients regardless of insurance status.

### 2.2. Remote Monitoring Intervention

For patients who agreed to be in the RM arm, there were two nursing visits to obtain consent, assess patient baseline parameters, and establish competency with technology. Physician orders were obtained before the nurse arranged a home visit. During the first visit, the visiting nurse obtained informed consent and instructed the patient and family about using the RM equipment. The second visit occurred within one week to make sure that the patient was comfortable using the technology and understood the procedures. Enrolled patients were taught how to measure their vital signs and weight, and transmit the readings to the RM nurse (a registered nurse practitioner with experience in management of cardiac patients). The RM nurse made weekly phone calls to the patients to provide additional instruction as needed, monitor adherence to the RM program, and solicit patient feedback about the program. Patients were instructed to transmit monitor readings including weight, blood pressure, pulse, and pulse oximetry on a daily basis. The Remote monitoring equipment included VitelNet, FDA-approved devices: a UA 767PC Turtle 400 monitor monitoring, a LifeSource digital weight scale, an A&D blood pressure/pulse cuff and meter, and a BCI pulse oximeter device (UC-321PBT). Patient data were transferred securely via telephone service to the Internet. They also answered a set of symptom-related questions including changes in shortness of breath or swelling. 

Vital ranges were established for each patient in consultation with their physician. If the readings received were outside the range expected for the patient, the RM nurse telephoned the patient to discuss the results. Increase in body weight was particularly emphasized as it is a strong predictor of hospitalization [15, 16]. The skilled nursing portion of the telephone call included evaluation of the RM readings and a telephone assessment of the patient. The RM nurse further evaluated patients who experienced clinical signs or symptoms of a worsening condition. Nurse recommendations included increased diuretic dose (if a physician's order was in place), physician or cardiologist notification, referral to the ER, and continued monitoring. To assess the patients' perceptions of the RM experience, patients were mailed surveys to complete at the end of the 6 month intervention. Questions for the satisfaction surveys were taken from previously validated surveys [17, 18]. New questions were added about technology perception ease of equipment use, program satisfaction, and option for open comments from patients. The final survey tool was tested during the study design phase but was not validated or checked for reliability.

### 2.3. Data Collection

Data on baseline information, previous and new readmissions, and other outcomes were collected through chart reviews by a physician. Any uncertainty regarding cause of readmissions was referred to an independent physician for arbitration. In addition to hospital records, we ascertained six-month mortality on study patients through the Social Security Death Index [19].

### 2.4. Statistical Analysis

The primary outcome for the study was all-cause re-hospitalization rate, determined by dividing total number of readmissions by the number of patients in each group. Secondary endpoints included re-hospitalization rate for HF, mortality, ER visits, length of stay, and participant satisfaction. We treated readmission rates as a continuous measure because patients with HF often experience multiple hospitalizations over a six-month period. Average length of stay was calculated by dividing the total number of inpatient days by the number of admissions in each group. All rates were compared between the three arms of the study: control, participant, and the non-participant group during an interim analysis at three-month stage and at the end of the six months. Differences in primary and secondary outcomes between the control and RM group were also compared using intention-to-treat analysis in which all patients offered RM, whether or not they agreed to RM, were analyzed together (Figure 1). Baseline variables and outcomes were compared between the groups using ANOVA for continuous variables and Fischer exact test for categorical variables. The Wilcoxon rank sum test was used for estimating readmission rates, ER visits, and Length of stay. All analyses were performed using SAS (SAS V.9 Cary, North Carolina) statistical software package.

## 3. Results

A total of 150 eligible subjects were identified over the course of one year and randomly assigned to control (*n* = 68, mean age = 70 ± 1.7 years) or RM group (*n* = 82, mean age = 66 ± 2.2 years). Of the 82 patients who were offered RM, 40 patients did not participate (patient refused = 24, physician refused = 16). Patients most commonly declined to participate because they were too busy, unsure of the technology, or worried that monitoring would make them feel disabled. Physicians who refused on behalf of their patients most frequently cited dislike of technology, fear of information overload, and doubt that their patient would cooperate. 

A summary of participant baseline characteristics and comorbid conditions by study arm is included in Table 1. The study sample had 44% females and it was a predominantly white population, above the age of 65 years. A majority of the patients were on Medicare, Medicaid, or other state insurance (77%). The three study arms were comparable for common comorbid conditions, cardiac medications, and ejection fraction. Excluding the index admission, patients had on an average less than one admission or ER visit due to HF in the previous year. 

Within 30 days of index admission, there were seven readmissions in control group, four readmissions in the RM intervention, and six readmissions in the non-participants. Participants in the RM program had a lower mean all-cause readmission rate (mean = 0.64, SD ± 0.87) compared with control (mean = 0.73, SD ± 1.51) and non-participant (mean = 0.75, SD ± 1.05) groups although this did not reach statistical significance (*P*-value = .75). The rate of HF-related readmissions was also similarly lower in the RM group (mean = 0.19, SD ± 0.45) compared to the control group (mean = 0.38, SD ± 1.06; *P* = .56) (Table 2). Interim analysis at the end of three-month stage had shown similar trends. Inpatient length of stay was shorter for the RM group as compared to non-participant and control groups (Table 2). All-cause ER visits, however, were higher in the RM group (0.83, SD ± 1.08) compared to the control group (0.57, SD ± 1.43; *P*-value = .10) which may be the result of patients more frequently reporting to the ER because of closer monitoring. An intention-to-treat analysis did not alter the results or the trends seen in the main analyses (Table 3). 

A total of 11 patients (four patients in control; four in RM; three in non-participants) died during the six-month period. Additionally, four patients in the RM group did not complete the full length of the program. Of the four, two patients moved to another city and were then not cared for by a Partners physician, and two stopped sending readings despite repeat phone calls from the RM nurse. For these subjects, all events up until the time of censoring were accounted for in the main analysis. The overall trends were not affected when the analysis was repeated leaving out subjects who died or did not complete the program.

On completion of the program, 20 of 42 subjects in the RM arm returned the satisfaction survey (response rate 48%). All these participants reported high level of satisfaction, with 93% respondents agreeing that the equipment was easy to use; the program improved their HF control; the program helped them stay out of hospital. All (100%) respondents reported that the equipment was simple and easy to use and the program made them feel more in control of their health. The majority of respondents (80%) also believed that the program should continue longer and was further supported by open comments such as “excellent opportunity to become more aware of my disease condition” and “with the program I have a tendency to be diligent about my diet and weight.”

## 4. Discussion

This pilot study demonstrated a trend towards a lower all-cause readmission rate in the RM group (0.64) doing better than the control (0.73), who in turn did better than the non-participant (0.75) groups. RM participants also had lower HF-related readmissions compared with the control group. We observed high level of patient satisfaction (93%) among RM participants and barriers to uptake of this technology were identified among non-participants. Thus our study demonstrates that not only can RM be successfully employed to deliver followup care, but also extending its use to a larger population may be potentially of great value to both patients and providers. 

As this is a pilot study that was not powered to demonstrate significant differences between groups in important HF-related endpoints, it is not surprising that the rates of these endpoints that we observed were not statistically different. The successful implementation of the RM program, the high degree of patient satisfaction, and the trends do suggest that RM may reduce HF hospitalizations and this certainly warrants further study. Our RM program was designed to prevent HF-related readmissions and it may have had less impact on all-cause readmissions (our primary outcome) for diverse reasons in the short period of study. There is also high comorbidity in HF patients. The event rate we observed in ambulatory patients is also much lower than that of homebound patients as demonstrated by the fact that patients in our pilot had on an average less than one HF-related readmission and ER visit in the year prior to index hospitalization (see Table 1). As such, the pilot's moderate sample size and short duration of followup (6 months) may have not allowed capture of sufficient events for trends to reach statistical significance. However, this pilot study indicates the value of studying the use of RM in a larger ambulatory population. Large-scale randomized trials are currently underway to elucidate the role of RM in HF management [20]. 

National efforts to disseminate RM approaches are growing. The Department of Veterans Affairs and various managed care organizations now use RM to care for patients with a variety of chronic conditions. A Veterans Affairs study demonstrated that the total number of inpatient hospital days for HF patients receiving RM fell from 630 for the previous year to 122 over the duration of the program [21]. The Centers for Medicare & Medicaid Services (CMS), an agency of the U.S. Department of Health and Human Services, is targeting readmissions to the hospital within 30 days of discharge as a probable marker for poor quality and efficiency of care [22]. Medicare has indicated that they may change payment policies that would result in hospitals with high risk-adjusted rates of readmissions receiving lower average per case payments than they do now. The specter of reimbursement cuts has stimulated some healthcare systems to invest in strategies to lower readmission rates. Remote monitoring has been identified as one of the potential intervention that can reduce avoidable readmissions. Our study provides evidence that the benefit of RM can be extended to the larger population of non-homebound patients traditionally excluded from such interventions.

Our findings also support previous studies that have shown benefits from RM, although the extent and nature of effects has varied across different outcomes [23–32]. Our study contributes to the literature by demonstrating the benefit of RM to ambulatory patients traditionally excluded from such programs. It was designed to additionally show feasibility, identify barriers to RM uptake in non-homebound patients, ascertain patient satisfaction, and provide data on event rates to guide future, larger trials. Our pilot trial has some important limitations. The study has a small sample size and was not powered to evaluate for differences in important clinical endpoints. We cannot ascertain which aspects of the RM program (weekly telephone calls, regular transmission of data, education, etc.) lead to the observed benefits. In addition, some aspects of the service may be time-consuming to deliver but add little to the effectiveness of the program. There are currently no standard methods for evaluating such programs, or agreement around standard definitions. This makes it difficult to decisively measure the impact of technology on delivery of care. Although we prospectively captured all hospitalizations and mortality events in a rigorous and complete manner, cost data and out-patient visit data were not fully ascertained. Cost analysis would be important especially if RM increases ER visits as happened in our study. Our qualitative surveys, although very positive, had modest completion rates (48%). The survey questions have been taken from prior studies but were not validated and were collected only at the end of the 6 months. Hence they may not accurately reflect change in perceptions or quality of life over a longer-term period.

Despite these limitations, the results from our pilot are sufficiently encouraging to warrant a larger randomized trial of RM technology within an ambulatory patient population. Our program found strong support from patients and their physicians who participated in the intervention and expressed their interest in continuing the program. We are currently offering a modified version of this program across all Partners hospitals in the Greater Boston area; to date over 300 patients have participated. Our modifications are based on the feedback received from participating patients and physicians, and we have addressed several barriers that were identified in the pilot trial. Current improvements include the development of a shared portal that allows more efficient communication between the RM nurse and the patient's care team, increased use of orders that allow the RM nurse to make timely treatment changes that have been approved by the patient's physician, identification of physician champions who promote the program amongst their colleagues, creation of a patient video to help prospective patients understand what the program involves and hear positive reactions from past participants, and use of an opt-out system where patients are enrolled unless a physician expressly declines thus increasing the proportion of eligible patients who get enrolled. We believe that these measures will enhance both the uptake and effectiveness of the overall program. 

In our pilot, 48% of the patients offered RM did not participate because either they or their physician refused. Implementation of the changes described above has lowered the refusal rate to 10%. The non-participant group remains of considerable interest to us because in our pilot they had the worst outcomes in terms of readmissions and ER visits. Ongoing efforts need to be made to overcome barriers to adoption in order that these patients can realize the benefits of RM. Although technology costs have fallen considerably over time, RM programs are likely to remain a limited resource and its use should be prioritized in candidates at highest risk for HF rehospitalizations. Adoption of a standardized methodology and framework for evaluation of these programs would be important for comparing different RM programs and identifying key features that promote success and the patient segments most likely to benefit. 

In conclusion, our pilot trial has demonstrated that the use of RM is a promising approach that has potential to reduce morbidity and increase patient satisfaction in non-homebound HF patients. Further investigation is warranted to determine how RM can be effectively implemented to optimize HF outcomes.

##  Funding 

The study was funded by Partners Healthcare. Partners Healthcare is a nonprofit integrated Health system founded by Brigham and Women's Hospital and Massachusetts General Hospital.

##  Disclosures 

The investigators were responsible for the study design, data collection, data analysis, data interpretation, and submission of the manuscript for publication, independently of all funding sources. The authors declare that they have no financial conflicts of interest to disclose.

## Figures and Tables

**Table 1:** Baseline characteristics and last one-year admissions.

Characteristics	Control (*n* = 68)	Intervention participants (*n* = 42)	Intervention non-participants (*n* = 40)	*P*-value*
Male gender (%)	64.7	61.9	45	.13
White race (%)	90	83.5	87.5	.64
Age, y, (mean ± SD)	70.2 (±1.7)	65.0 (±2.2)	67.9 (±2.3)	.2
Depression (%)	17.6	11.9	17.5	.71
Hypertension (%)	73.5	64.3	70	.58
Diabetes (%)	48.5	40.5	55	.42
Creatinine mg/dl (mean ± SD)	1.54 (±0.77)	1.51 (±0.86)	1.58 (±0.87)	.92
Ejection Fr. (mean ± SD)	0.37 (±0.18)	0.39 (±0.23)	0.42 (±0.21)	.51
No. of Cardiac meds^†^	6.2 (±0.24)	6.1 (±0.31)	5.7 (±0.32)	.41
Total no. of meds	11.6 (±0.55)	11.5 (±1.04)	10.8 (±0.21)	.64

Readmissions per patient in last 12 months prior to index hospitalization (mean ± SD)				

All cause	1.17 (±0.17)	0.75 (±0.22)	1.0 (±0.23)	.34
HF-related	0.8 (±1.04)	0.7 (±1.04)	0.6 (±0.69)	.78
ER visits (mean ± SD)				
All cause	1.34 (±1.47)	1.26 (±1.99)	1.65 (±1.83)	.16
HF-related	0.5 (±0.76)	0.57 (±1.34)	0.9 (±0.90)	.007

*(*P*-value = .05). ^†^Cardiac medications included Anticoagulants, vasodilators, digitalis, statins, diuretics, antiplatelet agents, angiotensin II receptor blockers, ACE-Inhibitors, Beta blockers, and calcium channel blocker.

**Table 2:** Six-month followup readmission rates and ER visit rate.

Hosp. readmissions (mean ± SD)	Control (*n* = 68)	Intervention Participants (*n* = 42)	Intervention non-participants (*n* = 40)	*P*-value*
All cause	0.73 (±1.51)	0.64 (±0.87)	0.75 (±1.05)	.75
HF-related	0.38 (±1.06)	0.19 (±0.45)	0.42 (±0.93)	.56
ER visits (mean ± SD)				
All cause	0.57 (±1.43)	0.83 (±1.08)	0.65 (±1.0)	.1
HF-related	0.25 (±1.02)	0.26 (±0.49)	0.35 (0.80)	.31
Length of stay (mean ± SD)				
All cause	10.64 (±9.7)	9.16 (±9.00)	13.2 (±13.4)	.85
HF-related	8.52 (±8.3)	10.57 (±12.5)	10.78 (±9.1)	.78

*(*P*-value = .05).

**Table 3:** Six-month results by Intention to Treat.

Hosp. readmissions (mean ± SD)	Control (*n* = 68)	Intervention^†^ (*n* = 40)	*P*-value*
All cause	0.73 (±1.51)	0.69 (±0.96)	.46
HF-related	0.38 (±1.06)	0.30 (±0.73)	.5
ER visits (mean ± SD)			
All cause	0.57 (±1.43)	0.74 (±1.04)	.06
HF-related	0.25 (±1.02)	0.30 (±0.66)	.12
Length of stay (mean ± SD)			
All cause	10.64 (±9.7)	11 (±11.34)	.96
HF-related	8.52 (±8.3)	10.68 (±10.36)	.55

*(*P*-value = .05). ^†^Intervention includes all patients who were offered the opportunity to participate.
